# A descriptive analysis of the contents of Care Response, an international data set of patient-reported outcomes for chiropractic patients

**DOI:** 10.1186/s12998-023-00509-w

**Published:** 2023-09-19

**Authors:** Kenneth J Young, Jane Fitzgerald, Jonathan Field, David Newell, Jim Richards

**Affiliations:** 1 University of Central Lancashire, Preston, UK; 2 University of Southampton, Southampton, UK; 3 AECC University College Bournemouth, Bournemouth, UK

**Keywords:** Chiropractic, Patient-reported outcome measures, Database, Descriptive analysis

## Abstract

**Background:**

Databases have become an important tool in understanding trends and correlations in health care by collecting demographic and clinical information. Analysis of data collected from large cohorts of patients can have the potential to generate insights into factors identifying treatments and the characteristics of subgroups of patients who respond to certain types of care. The Care Response (CR) database was designed to capture patient-reported outcome measures (PROMs) for chiropractic patients internationally. Although several papers have been published analysing some of the data, its contents have not yet been comprehensively documented. The primary aim of this study was to describe the information in the CR database. The secondary aim was to determine whether there was suitable information available to better understand subgroups of chiropractic patients and responsiveness to care. This would be achieved by enabling correlations among patient demographics, diagnoses, and therapeutic interventions with machine learning approaches.

**Methods:**

Data in all available fields were requested with no date restriction. Data were collected on 12 April 2022. The output was manually scanned for scope and completeness. Tables were created with categories of information. Descriptive statistics were applied.

**Results:**

The CR database collects information from patients at the first clinical visit, 14, 30, and 90 days subsequently. There were 32,468 patient responses; 3210 patients completed all fields through the 90 day follow up period. 45% of respondents were male; 54% were female; the average age was 49. There was little demographic information, and no information on diagnoses or therapeutic interventions. We received StartBack, numerical pain scale, patient global impression of change, and Bournemouth questionnaire data, but no other PROMs.

**Conclusions:**

The CR database is a large set of PROMs for chiropractic patients internationally. We found it unsuitable for machine learning analysis for our purposes; its utility is limited by a lack of demographic information, diagnoses, and therapeutic interventions. However, it can offer information about chiropractic care in general and patient satisfaction. It could form the basis for a useful clinical tool in the future, if reformed to be more accessible to researchers and expanded with more information collected.

**Supplementary Information:**

The online version contains supplementary material available at 10.1186/s12998-023-00509-w.

## Background

Advances in digital information management systems, including databases, have become important tools in understanding trends and correlations in health care by improving the collection and organisation of information such as demographics, diagnostics, therapeutic interventions, and outcomes. Routine collection of such data has now become commonplace in national health systems like the United Kingdom National Health Service (NHS) with recent imperatives focusing on the most efficient use of such data for key goals such as, improving patient outcomes, increased efficiency, and the development of effective new treatments [1].

Analysis of data collected from large cohorts of patients can have the potential to generate insights into factors identifying treatments and the characteristics of subgroups of patients who respond to certain types of care. Observational studies using large data sets have led to important public health discoveries through epidemiological analysis. For example, the Framingham study led to landmark breakthroughs in the understanding of blood pressure in disease [2].

The advent of machine learning (ML) techniques, a form of artificial intelligence (AI), is starting to change health care in profound ways, including reducing bias in diagnosis, facilitating the use of multiple sources of information to improve the understanding of pathological conditions, and improving the analysis of data sets [3]. [4] ML techniques are derived from traditional statistical methods and do not always provide clarity when trying to understand complex systems [4]. However, ML is beginning to reduce human burden in the task of diagnosis in a world of increasingly complex and interacting factors [4].

Computer technology has also made it easier to create databases that can be tailored for specialised purposes. The Care Response (CR) database has existed for ten years now and has grown steadily over time. It was designed to capture patient-reported outcome measures (PROMs) for chiropractic patients internationally. Different PROMs capture information in one or more of five different categories: quality of life related to health, functional status, symptoms, health behaviours, and patients’ perspective on their health care experience [5, 6]. PROMs can be used to monitor patient progress, guide clinical decisions, and benchmark treatment outcomes [7, 8] The use of PROMs has been associated with improved communication between clinician and patient, better symptom control, and increased patient satisfaction [9].

Specifically, the CR database was developed to help clinicians overcome some of the barriers to using PROMs regularly in day-to-day clinical practice. Electronic PROM systems are believed to reduce the administrative burden (time & cost), simplify the collection and reporting of results as well as increase the completeness of returned assessments [5, 10–13] PROMs available on the CR system include the Bournemouth Questionnaire (BQ), Measure Your own Medical Outcome Profile (MYMOP), EQ-5D, patient global impression of change (PGIC), numerical pain scale, and Patient Reported Experience Measure (PREM) [14].

The BQ was developed to be a quick, comprehensive assessment instrument. Studies have found the BQ to be valid and reliable in back [15] and in neck [16] pain patients. MYMOP [17], is a health-related quality of life (HRQL) measure. It allows patients to nominate and score the two most important aspects of their lives that contribute most to their overall quality of life. The EuroQol 5 dimensions questionnaire (EQ-5D), is also an HRQL measure. It is commonly used for people in chronic pain [18]. Compared to some other measures, the EQ-5D has produced higher scores in healthier people, and lower scores in severely ill patients. The EQ-5D may also be more sensitive in conditions with primarily physical limitations and disability [19]. The PGIC is a 7-point ordinal Likert scale (very much improved, much improved, minimally improved, not changed, minimally worse, much worse and very much worse) [20]. The numerical pain scale (NPS) is an 11-point (0–10), commonly used pain rating tool which is very quick to administer but it is challenging to use to infer clinically important changes from baseline [21]. The PREM in the CR database is two open-ended questions: (1) Is there anything particularly good about your chiropractic care? and (2) Is there anything that could be improved?

The CR database is currently provided to clinicians as a free-to-use online system. Designed to be used in busy clinics, the advantage of the CR database is that it moves away from reliance on clinicians to provide PROMs themselves during consultations, collecting information directly from patients. After obtaining informed consent, clinics register patients by adding their name, date of birth, email address and date of first appointment either using a ’self-service’ link provided by the clinic or by clinic administrative staff. Once these fields are populated, the CR database generates a PROM questionnaire, based on the clinician’s preference, and this is provided to the patient usually via an automated email link or by the clinic on a PC or tablet device, or in paper form. Any of the available PROM instruments can be selected as a whole; questions cannot be selected from different PROMs and combined by a clinician. Multiple PROMs may be selected for use by any clinic. Subsequent PROM questionnaires are generated either at pre-set timed intervals or by the clinic manually making a request. PROM questionnaires are scored, and the clinician is presented with collated results for an individual patient or group of patients in tabulated or graphic format for longitudinal assessment after serial use of PROMs. The results of these are available immediately they are completed facilitating their use within a clinical encounter.

The CR database has been adopted in clinical practice across diverse settings and multiple countries and 218,770 patients used the CR database up to September 2022. However, up to date information on the number of clinics or countries is not available, as the system is anonymised. There are also no data on the numbers of NHS versus private patients.

Data from the CR database have been used to support multiple publications, including one of the largest UK wide cohort studies within the chiropractic profession to inform the feasibility of online PROM collection [22]. Others include the use of PROMs in clinical education, [23] studies on outcomes [24, 25] and predictors of response to chiropractic care [26, 27] There has also been a series of papers exploring the utility of the STarT Back Screening tool in professions working in the independent sector [28–30]. Care Response data have supported 2 PhD studies, including one which resulted in the development of a theoretical model of the impact of PROMs in clinical practice [31, 32] But despite these studies there has not yet been a comprehensive assessment of the contents of the CR database.

We sought detailed participant demographics, signs and symptoms, diagnostic data as well as information on therapeutic interventions, length of treatment sessions and length of care plans. We hoped in a future study to apply ML methods to the large number of variables in the large cohort of participants to glean insight into which interventions were most effective for which conditions. This could then support clinical decision-making and policy development [33]. With information on signs and symptoms, it may be possible to discover previously undiagnosed conditions [34].

This study therefore aimed to generate a description and preliminary analysis of the entire data record, with the following objectives: to document the content of the CR database, and to explore whether the depth and breadth of information contained within the CR database would be appropriate for the application of ML analysis.

## Methods

Prior to commencing data collection, data sharing agreements were drawn up between the University of Central Lancashire (UCLan) and AECC-University College (AECC-UC), and between UCLan and Clinical Transparency, Ltd, owned by Jonathan Field, which holds the CR database. Ethical approval was obtained through the UCLan Research Ethics Committee, approval #HEALTH0287. Consent to participate is obtained by Care Response and includes the option of use for research by third parties.

Two authors (KJY and JFitzgerald) first manually scanned the data to assess a broad sense of scope and completeness. The search was not date-limited, including the full 10 + years of data in the CR database. Categories of data, such as demographics, were extracted into separate tables to facilitate analysis. Without knowing what data were collected by the CR system, we were unable to determine a priori which data points we would analyse. We broadly considered that we would explore the association of demographic factors and response/completion rates. We did not know if there may be differences in responsiveness to individual items, therefore we included individual items in the analysis. Descriptive statistics were calculated for timepoints based on relevant patient characteristics available in the dataset. Based on the contents of the database, a judgement was made on the suitability of ML analysis.

## Results

Anonymised data were extracted from the database on 12 April 2022. After data extraction from the CR servers, we received an Excel spreadsheet with 32,468 rows, each with 95 columns. 30,940 people (95%) completed the baseline data; 14,695 (45%) completed the 14 days post-clinical-visit questions; 12,764 (39%) completed at 30 days, and 8689 (26%) completed at 90 days. These figures do not reflect completion of all questions up to these time points; that is, for instance, someone could have completed the baseline and only the 90-day questions.

The database contains clinical metrics including various validated PROMs. A list of available data points was obtained which included patient age and sex, region of pain, patient global impression of change, pain rating, and information from the STarT Back Screening tool [35] and Bournemouth Questionnaire [15, 16]. Please see Additional File 1 for a list of the available data points and their codes and further details. However, other variables were limited and did not include information on signs and symptoms, diagnostic data, therapeutic interventions, length of treatment sessions or length of care plans. There were no data returned relating to the EQ-5D, MYMOP, or PREM outcome measures. Of the 32,468 rows 3,210 contained a complete data set, that is, every box filled in, to 90 days. When patients sign up to the system, they are given an option to have their anonymised data used for analysis and only those who gave this consent were included in the data export. That proportion of the total was unknown.

**Table 1:** Timepoint Completion – numbers of people who completed input at the different points sampled

	All records		Male		Female		Unknown Gender	
Baseline/14/30/90 Day Completion	All	%	All	%	All	%	All	%
**Total Entries**	32,468		32,468		32,468		32,468	
Complete Baseline	30,491	93.91	13,950	42.97	16,722	51.50	264	0.81
Complete Baseline AND Complete 14 days	13,846	42.65	5758	17.73	7978	24.57	108	0.33
Complete Baseline AND 14 AND 30 days	8463	26.07	3429	10.56	4976	15.33	56	0.17
Complete Baseline AND 14 AND 30 AND 90	5092	15.68	2107	6.49	2956	9.10	27	0.08

Table 1 shows participants who completed the Baseline, 14 days, 30 days and 90 days; 15.65% of those who completed all database fields completing through to 90 days. It is of note that more female patients completed through to 90 days than male patients, but the data gave no indication of why this might be.

**Table 2:** Patient characteristics

	All records				Male				Female			
Demographics	All	%	Complete	%	All	%	Complete	%	All	%	Complete	%
**Total Entries**	32,468	100.00	3210	9.89	32,468		3210	9.89	32,468		3210	9.89
Male/Female Records					14,535	44.77	1396	43.49	17,631	54.30%	1802	56.14
Average Age	49	N/A	51	N/A	49	N/A	53	N/A	49	N/A	50	N/A
New to the Practice	4585	14.12	677	21.09	2188	6.74	298	9.28	2395	7.38%	379	11.81
Seen same Practitioner before	2996	9.23	398	12.40	1494	4.60	190	5.92	1501	4.62%	208	6.48

Table 2 was created to explore whether there may have been a link between completion of data points and data such as gender, age, new to practice and same therapist; once again, the completion by female patients was higher than that of male patients. Age was indistinguishable across both genders and completion data.

**Table 3:** Responses to STarT Back Screening Tool (SBT)

SBT Average	All			Complete		
	All	Male	Female	All	Male	Female
Leg Pain	0.49	0.47	0.51	0.49	0.47	0.51
Neck Pain	0.56	0.49	0.61	0.56	0.49	0.62
Short Walk	0.46	0.43	0.48	0.46	0.42	0.48
Dressing	0.60	0.63	0.57	0.60	0.63	0.57
Safe Activities	0.25	0.26	0.23	0.25	0.26	0.23
Worrying	0.36	0.36	0.35	0.36	0.36	0.35
Never Improves	0.51	0.51	0.51	0.50	0.50	0.51
Not enjoying things	0.62	0.61	0.62	0.62	0.61	0.62
Bothersomeness	2.75	2.69	2.80	2.74	2.69	2.79

Table 3 shows the responses to the STarT Back Screening tool in terms of average pain scores, with little gender difference seen except possibly in “bothersomeness”. However, we did not test whether this difference may be significant or not, as we were only seeking to document the contents and completeness of the database at this time.

**Table 4:** STarT Back Ranking responses

SBT Ranking	All Records						Complete Records					
216 unknown gender	All	%	Male	%	Female	%	All	%	Male	%	Female	%
No of Records	32,468		14,535		17,636		21,672		10,573		12,708	
Low	8605	0.27	4000	0.28	4523	0.26	7912	0.37	4000	0.38	4523	0.36
Medium	8708	0.27	3772	0.26	4849	0.27	7654	0.35	3562	0.34	4602	0.36
High	6719	0.21	3047	0.21	3621	0.21	6106	0.28	3010	0.28	3582	0.28
Incomplete	6908	0.21	3131	0.22	3733	0.21						
Blank	1527	0.05	585	0.04	909	0.05						

Table 4 compared the level of pain expressed by patients, considering data completion and gender; the highest scores were for low and medium pain, with a slight increase in the “low” sector. Table 5 shows the responses to the NPS. Table 6 reviews the average scores across the pain domains, around 6/10 at baseline and reducing to an average of 2.15, with those completing all time points showing a lower pain score than those who didn’t complete all data points.

**Table 5:** Responses to numerical pain scale (0–10)

			Male		Female	
Pain	All	Complete	All	Complete	All	Complete
Average Days since month w/o pain	377.34	375.13	453.25	343.03	399.67	496.77
Average Pain rating at baseline	6.41	6.33	6.26	6.15	6.53	5.86
Average Pain rating at 14 days	4.33	3.79	3.98	3.48	4.58	4.02
Average Pain rating at 30 days	3.70	2.89	3.33	2.59	3.95	3.11
Average Pain rating at 90 days	3.35	2.70	3.01	2.46	3.61	2.88

**Table 6:** Average Bournemouth questionnaire information

		Complete		Male		Female	
	Bournemouth Scale	All	Complete	All	Complete	All	Complete
Baseline	Pain	6.41	6.33	6.26	6.15	6.53	6.47
	ADL	5.42	5.66	5.31	5.40	5.50	5.87
	Social	4.92	5.11	4.97	5.00	4.86	5.20
	Anxiety	4.69	4.70	4.56	4.33	4.79	4.99
	Depression	3.48	3.41	3.25	3.01	3.65	3.70
	Work	4.99	5.05	4.89	4.72	5.06	5.31
	Locum of Control	4.90	5.10	4.84	4.90	4.94	5.25
	Total	34.77	35.37	34.05	33.52	35.31	36.79
14 Days	Pain	4.33	3.79	3.98	3.48	4.58	4.02
	ADL	3.26	3.10	3.01	2.79	3.44	3.33
	Social	3.03	2.90	2.91	2.72	3.12	3.05
	Anxiety	3.08	2.88	2.84	2.54	3.26	3.14
	Depression	2.41	2.19	2.23	1.94	2.53	2.39
	Work	3.30	3.17	3.07	2.90	3.46	3.39
	Locum of Control	3.18	3.02	3.02	2.84	3.29	3.17
	Total	22.60	21.04	21.06	19.21	23.69	22.48
30 days	Pain	3.70	2.89	3.33	2.59	3.95	3.11
	ADL	2.77	2.30	2.52	2.06	2.94	2.49
	Social	2.56	2.13	2.43	2.00	2.64	2.23
	Anxiety	2.66	2.25	2.40	1.93	2.84	2.50
	Depression	2.16	1.80	1.92	1.52	2.32	2.01
	Work	2.86	2.45	2.66	2.19	3.00	2.65
	Locum of Control	2.73	2.35	2.57	2.16	2.83	2.50
	Total	19.43	16.16	17.84	14.45	20.53	17.49
90 days	Pain	3.35	2.70	3.01	2.46	3.61	2.88
	ADL	2.46	2.17	2.22	1.96	2.63	2.34
	Social	2.29	2.00	2.13	1.85	2.40	2.11
	Anxiety	2.48	2.22	2.19	1.93	2.69	2.45
	Depression	2.05	1.83	1.83	1.58	2.20	2.02
	Work	2.54	2.29	2.32	2.08	2.69	2.46
	Locum of Control	2.48	2.24	2.32	2.07	2.58	2.37
	Total	17.64	15.44	16.02	13.92	18.80	16.64

**Table 7:** Patient Global Impression of Change (PGIC)

			Male		Female	
PGIC - Patient Global Impression of Change	All	Complete	All	Complete	All	Complete
Average total at 14 days	5.24	5.51	5.25	5.57	5.07	5.47
Average total at 30 days	5.49	5.88	5.58	5.95	5.43	5.84
Average total at 90 days	5.57	5.88	5.65	5.90	5.52	5.86

Table 7 shows a difference in the impression of change in pain level between those who completed all data-points and those who did not. Table 8 shows a marked improvement in satisfaction between those who completed all the data points and those who did not.

**Table 8:** Patient satisfaction Only data from 30 days post appointment was retrieved. Scale is 0–10 with 10 representing complete satisfaction

Satisfaction	All			Complete		
	All	Male	Female	All	Male	Female
Satisfaction at 30 days	5.86	5.86	5.87	6.03	6.23	6.27

## Discussion

We found the CR database somewhat limiting to use for clinical research. It was designed for practitioners to better understand their practices, and so perhaps unsurprisingly other uses require workarounds. Member clinics get reports on their PROMs free of charge, but there was an additional financial cost to extract the data for the current analysis. Compared to other databases (e.g. healthdata.gov, healthdata.org.uk, Cochrane Open Access), the information in the CR database was limited.

The CR database anonymises responses and so tracking of individual patients was not possible. One patient may be represented by multiple lines, so this is a major limitation. Many patients could have a few rows each and thus the data could quickly be greatly biased. We also have no indication that either responders to care or non-responders would be more likely to participate in CR. Care Response did not include any information on diagnosis, co-morbidities, type of treatment interventions used by the reporting clinic, the number of visits for treatment, or co-treatment by other practitioners. The demographic information included only sex and age. These are points that could be addressed in a future version of CR.

Studies have shown that chiropractic care often produces improvements in clinical outcomes, particularly for low back pain [36–38] The generally positive outcomes found in the CR database could be the result of the therapeutic interventions applied. However, the therapeutic alliance and contextual factors are also emerging as important elements in clinical improvement [39–43] There is evidence that chiropractors develop a positive therapeutic alliance with their patients [44]. Therefore, it seems likely that improved clinical outcomes are due to these effects in various combinations and proportions. However, it should be noted that regression to the mean, expected improvement over time due to natural history, and other factors are also likely involved in producing these results.

The data currently available on the CR database are limited. The available data are not useful in terms of helping to determine which therapeutic interventions correlate with better scores on PROMs, which interventions are reported as more effective on which conditions or which demographic groups. Without information on diagnoses and therapeutic interventions, we identified a lack of utility in the current state of the CR database to use ML or statistical methods for clinical prediction.

There are positive elements to the CR database. It is one of few large cohort databases within the realm of chiropractic. Generally, large chiropractic cohort studies have focused on specific research questions [45–49] There is no other system that has broadly collected PROMs and PREMs information on chiropractic patients on an ongoing basis for over 10 years and continuing now. The CR database is not limited to one country, and thus researchers in the future could design studies to compare outcomes in different international regions. Finally, patients can enter information directly into the system electronically via the internet. As noted above, this may facilitate response rates and reduce the time clinic staff use to collect the data.

### Strengths of this study

This is the first study to explore the contents of the CR database for its potential to be investigated using machine learning methods, although the result was ultimately negative. In addition, although a number of observational cohort studies of chiropractic patients can be found in the literature, they involved dozens to low thousands of participants, [50–55] whereas our analysis of the CR database contained PROMs data for over 33,000 individual courses of care, with the caveat that we do not know how many individual patients this represents. We view Care Response as augmenting the information in other large cohorts collecting data on chiropractic patients. So, understanding the scope and completeness of the data is useful to help guide future research projects using this database.

### Limitations

The sample group included in CR is limited to only those patients who chose to include themselves, attending clinics where the practitioners have chosen to enrol on the system. Consequently, the cohort would seem to include only the highly motivated patients of highly motivated practitioners. Thus, there is potential inclusion bias in the sample. The potential inclusion of patients on multiple lines on the spreadsheet of data was another potential source of bias. There was a lot of missing data; about 16% of patients completed all items through to the 90-day follow-up. It was unknown as to how many returning patients were also included as new patients, so there may have been some overlap or mis-categorisation of patients. Despite the large size of the data set, information on outcomes is not generalisable to chiropractic patients at large due to the amount of information missing or not collected.

### Further research

It is challenging to know how meaningful correlational analyses might be. Is it useful to know, for instance, if female patients, or an older age group responded better to ‘chiropractic care’? If the system collected more data about diagnostic and treatment information, as well as reporting location, socio-economic, and cultural data about patients, more conclusions could be drawn about which aspects of chiropractic care provide the most benefit to patients.

## Conclusions

The CR database is a large set of PROMs for chiropractic patients internationally. However, its utility is limited by a lack of demographic information, diagnoses, and therapeutic interventions. It can offer information about chiropractic care in general and patient satisfaction. It could form the basis for a useful clinical tool in the future, if reformed to be more accessible to researchers and expanded with more patient demographic data points as well as information on diagnoses and therapeutic interventions.

### Electronic supplementary material

Below is the link to the electronic supplementary material.

Supplementary Material 1

## Data Availability

Data will not be available due to the proprietary interests of Clinical Transparency, Ltd.
