# Supplementary material for: A descriptive analysis of the contents of Care Response, an international data set of patient-reported outcomes for chiropractic patients
Source: Chiropr Man Therap. 2023 Sep 19;31:37. doi: 10.1186/s12998-023-00509-w (PMC10510118; doi:10.1186/s12998-023-00509-w)
Supplement: Supplementary file 1 — Supplementary Material 1 [file 12998_2023_509_MOESM1_ESM.docx]

| Care Response Code | Meaning |
| --- | --- |
| CRPatientId | A unique identifier for the patient in the Care Response system |
| ClientFileNo | A unique identifier given to the patient by the clinic (optional field) |
| FirstAppointmentDate | Date of patient’s first appointment at that clinic |
| CreatedDate | Date of the first entry on the Care Response system for that patient |
| StopAssessement | Patient has requested to stop participating in the Care Response system |
| PresentationId | If a patient presents with a new complaint, a new unique identifier is given for the condition |
| AgeAtFirstAppointment | Self-explanatory |
| Gender | Self-explanatory |
| DaysToFirstAppointmentDate | Patients may be entered on the system before they attend the clinic; this field indicates how many days that is. |
| PracticeId | Unique identifier given to the clinic by Care Response |
| PractitionerId | Unique identifier given to the practitioner by Care Response |
| IsNewPatient | Is the patient new to the practice? |
| HasSeenPractitionerBefore | Has the patient seen the same practitioner previously? |
| AssessmentCompletionMethod | How the patient’s information is entered on to the Care Response system (e.g. email, hard copy documents at a clinic) |
| IsThereHeadPain | Self-explanatory, entered at baseline |
| IsThereNeckPain | Self-explanatory, entered at baseline |
| IsThereShoulderPain | Self-explanatory, entered at baseline |
| IsThereBackPain | Self-explanatory, entered at baseline |
| IsThereArmAboveElbowPain | Self-explanatory, entered at baseline |
| IsThereArmBelowElbowPain | Self-explanatory, entered at baseline |
| IsTherePainLegThighKnee | Self-explanatory, entered at baseline |
| IsTherePainBelowKnee | Self-explanatory, entered at baseline |
| Diagnosis | A limited list is available, and this is an optional field |
| DaysSince30DaysOfNoPain | How long has it been since the patient had a full month without pain? |
| PainOver30daysInYear | Does the patient have pain for more than 30 days per year? |
| IsProblemReocurring | Self-explanatory |
| SBTRanking | All the “SBT” headers indicate questions from the STarTBack questionnaire |
| SBTLegPain | “ |
| SBTNeckPain | “ |
| SBTShortWalking | “ |
| SBTDressingSlowly | “ |
| SBTSafeActivity | “ |
| SBTWorryingThoughts | “ |
| SBTNeverImprove | “ |
| SBTNotEnjoyThings | “ |
| SBTBothersomness | “ |
| Pain | Numerical pain scale (0-10). The same question is used whenever Care Response asks about pain scale. This means that if a patient is not completing the Bournemouth Questionnaire (BQ) but another assessment questionnaire they will be asked the BQ question 1 to rank their pain. |
| BQADL | All the “BQ” headers indicate questions from the Bournemouth Questionnaire |
| BQSocial | “ |
| BQAnxiety | “ |
| BQDepression | “ |
| BQWork | “ |
| BQLocusOfControl | “ |
| BQbaselineTotal | “ |
| 14dDaysSinceAppt | The number of days since first appointment at clinic |
| 14dVisitsSoFarOUTCOME | Patient’s recollection of the number of visits they have had when completing the Care Response assessment 14 days after starting treatment. |
| 14dPain | Numerical pain scale (0-10), 14 days after first appointment, the first question on the Bournemouth Questionnaire. All the “BQ” headers indicate questions from the Bournemouth Questionnaire. |
| 14dBQADL | “ |
| 14dBQSocial | “ |
| 14dBQAnxiety | “ |
| 14dBQDepression | “ |
| 14dBQWork | “ |
| 14dBQLocusOfControl | “ |
| 14dBQbaselineTotal | “ |
| 14dPGIC | What is the Patient’s Global Impression of Change (PGIC) to their condition 14 days after their first appointment? |
| 30dDaysSinceAppt | The number of days since first appointment at clinic |
| 30dVisitsSoFarOUTCOME | Patient’s recollection of the number of visits they have had when completing the Care Response assessment 30 days after starting treatment. |
| 30dPain | Numerical pain scale (0-10), 30 days after first appointment |
| 30dBQADL | Numerical pain scale (0-10), 30 days after first appointment, the first question on the Bournemouth Questionnaire. All the “BQ” headers indicate questions from the Bournemouth Questionnaire. |
| 30dBQSocial | “ |
| 30dBQAnxiety | “ |
| 30dBQDepression | “ |
| 30dBQWork | “ |
| 30dBQLocusOfControl | “ |
| 30dBQbaselineTotal | “ |
| 30dPGIC | What is the Patient’s Global Impression of Change (PGIC) to their condition 30 days after their first appointment? The question was: “How would you describe your pain/complaint now, compared to how you were when you completed the questionnaire before your first visit to this clinic?” The scale ranges from 1 (worse than ever) to 7 (very much improved). |
| 30dSatisfaction | Patient’s satisfaction with their care 30 days after their first appointment, on a scale of 0 (worst) to 7 (best) The question was: “Overall, how have you found the service and care your received? This would include the way you have been treated by our reception, practitioners or any other contact from us. Please select one of the following”: (1) unacceptably poor; (2) not as good as I was expecting, I would be concerned if a friend wanted to come to you; (3) reasonable but nothing special; (4) as I was expecting and I am satisfied with this; (5) better than I was expecting; (6) good, I would be happy to  recommend to a friend to you; and (7) a very high level, I  would recommend friends with similar problems to consider. |
| 90dDaysSinceAppt | The number of days since first appointment at clinic |
| 90dVisitsSoFarOUTCOME | Patient’s recollection of the number of visits they have had when completing the Care Response assessment 90 days after starting treatment. |
| 90dPain | Numerical pain scale (0-10), 90 days after first appointment |
| 90dBQADL | Numerical pain scale (0-10), 90 days after first appointment, the first question on the Bournemouth Questionnaire. All the “BQ” headers indicate questions from the Bournemouth Questionnaire. |
| 90dBQSocial | “ |
| 90dBQAnxiety | “ |
| 90dBQDepression | “ |
| 90dBQWork | “ |
| 90dBQLocusOfControl | “ |
| 90dBQbaselineTotal | “ |
| 90dPGIC | What is the Patient’s Global Impression of Change (PGIC) to their condition 90 days after their first appointment? |
| NHS | Is the patient attending as part of an NHS service? |
| OK_Research_Contact | Does the patient give permission to be contacted for further research, beyond filling in Care Response questionnaires? |
| Contact_By_Telephone | What is the preferred method of contact, telephone? |
| Contact_By_Email | What is the preferred method of contact, email? |
| Contact_By_Post | What is the preferred method of contact, post? |

Legend: Our request for all possible data returned these fields, indicated by codes in an Excel spreadsheet (left column). Their meanings are in the right column. No data for the MYMOP, EQ5D, or PREM were returned.
